# Supplementary material for: MetaRibo-Seq measures translation in microbiomes
Source: Nat Commun. 2020 Jun 29;11:3268. doi: 10.1038/s41467-020-17081-z (PMC7324362; doi:10.1038/s41467-020-17081-z)
Supplement: Supplementary file 10 — Supplementary Data 7 [file 41467_2020_17081_MOESM10_ESM.zip › File2/Confidence_High_Taxonomy/223791_out.krona.html]

Javascript must be enabled to view this page.

members
magnitude
magnitudeUnassigned
count
unassigned
taxon
rank

223791\_out

8

8
2
superkingdom

phylum
8
1239

8
186801
class

186802
8
order

family
8
186803

33042
8
genus

species

SRS144297\_contig\_number\_1859
1263071
1

species

SRS1055022\_contig\_number\_14082
1
2049024

1
2293096

SRS893366\_contig\_number\_21516
species

5
33043
species

SRS014923\_contig\_number\_42367SRS050925\_contig\_number\_25755SRS098571\_contig\_number\_29824SRS101376\_contig\_number\_5516SRS144714\_contig\_number\_15378
